# Retinal microcirculation and leukocyte telomere length in the general population

**DOI:** 10.1038/s41598-018-25165-6

**Published:** 2018-05-04

**Authors:** Dries S. Martens, Fang-Fei Wei, Bianca Cox, Michelle Plusquin, Lutgarde Thijs, Ellen Winckelmans, Zhen-Yu Zhang, Tim S. Nawrot, Jan A. Staessen

**Affiliations:** 1 Centre for Environmental Sciences, Hasselt University, Hasselt, Belgium; 2 Studies Coordinating Centre, Research Unit Hypertension and Cardiovascular Epidemiology, KU Leuven Department of Cardiovascular Sciences, University of Leuven, Leuven, Belgium; 3 Department of Public Health & Primary Care, University of Leuven, Leuven, Belgium; 4 R&D Group VitaK, Maastricht University, Maastricht, The Netherlands

## Abstract

Retinal arteriolar narrowing increases with age and predict adverse cardiovascular outcomes. Telomere length keeps track of the division of somatic cells and is a biomarker of biological age. We investigated to what extent retinal arteriolar diameters are associated with biological age, as captured by leukocyte telomere length (LTL). In 168 randomly selected Flemish participants from the family-based population study FLEMENGHO (mean age, 46.2 years) at baseline, of whom 85 underwent a follow-up examination (median, 4.1 years), we post-processed nonmydriatic retinal photographs and measured LTL. In men only, central retinal arteriolar equivalents (CRAE) and arteriole-to-venule ratio (AVR) were associated with LTL with stronger associations at higher age and body mass index. In men aged 57.6 years (75th percentile) a 20% shorter LTL was associated with a decrease in CRAE of 4.57 µm. A 20% shorter LTL was associated with a decrease of 5.88 µm in CRAE at a BMI of 29.9 kg/m^2^ (75th percentile). Similar associations were observed between AVR and LTL. In women, no retinal microvascular traits were associated with LTL. Retinal arteriolar narrowing in men but not in women is associated with biological age. Our findings support the idea that avoiding overweight contributes to maintaining a healthier microcirculation.

## Introduction

Human ageing is a complex phenotypical trait that dynamically responds to environmental factors^1–3^. Chronological age is a major risk factor for cardiovascular disease. Telomeres are nucleoprotein structures of tandem TTAGGG repeats at the chromosome ends. They function as a protective cap for degradation and are necessary to maintain genomic stability^4^. Telomeres shorten with each cellular replication due to the end-replication problem and leukocyte telomere length (LTL) is considered a marker for biological age.

Changes in the retinal microvascular system occurs with ageing and may result from arteriolar damage due to hypertension^5^. Indeed increased ambulatory blood pressure is predictive for central retinal arteriolar narrowing (CRAE) after 10.3 years of follow up^6^. Arteriolar narrowing may be predictive of cardiovascular events and the diameters of the retinal microvessels may provide important prognostic information for macrovascular complications, organ function and damage^7^. A decrease in the retinal arteriole-to-venule ratio (AVR) has been associated with higher relative risks of stroke^8^, coronary heart disease and myocard infarction in women but not men^9^. Telomere length as a surrogate for biological age has been investigated as a predictor of macrovascular related conditions such as coronary heart disease^10^, hypertension^11,12^, atherosclerosis^13,14^, and stroke incidences^15^.

Whether retinal microvascular traits are related to LTL is currently unknown. We therefore assessed the association of retinal microvascular phenotypes with LTL in a random population sample of the prospective family-based population study FLEMENGHO (Flemish Study on Environment, Genes and Health Outcomes).

## Results

### Characteristics of participants

The study population consisted of 13 singletons and 155 related subjects, belonging to 21 one-generation families and to 20 multi-generation pedigrees. Table 1 provides baseline characteristics for the 168 participants by tertiles of LTL. All participants were white European and included 89 (53%) women. The mean age (±SD) was 46.2 ± 14.9 years at baseline and ranged from 19 to 79 years. 69 (41.1%) had hypertension and 9 (5.4%) were diagnosed with diabetes at baseline. Participants in the lowest category of LTL were older, had a higher BMI, had higher systolic and diastolic blood pressure and showed a higher prevalence of hypertension. The mean (±SD) CRAE and CRVE was 151.7 ± 15.2 μm and 218.5 ± 18.9 μm respectively and the mean AVR was 0.70 ± 0.06. Average relative LTL ranged from 0.55 to 1.61. Of the 168 participants, 85 had a second follow-up after a median of 4.1 (range: 2.7 to 4.8) years, 45 of them were men and 40 women.

**Table 1:** Characteristics of participants at baseline by tertiles of leukocyte telomere length.

Characteristic	Tertiles of average relative LTL (*n* = 168)			*P*-value
	<0.96	0.96–1.15	≥1.15	
Number of participants (%)	56	55	57	
Women	29 (51.8)	28 (50.9)	32 (56.1)	0.84
Smoker	9 (16.1)	11 (20.0)	6 (10.5)	0.38
Drinking alcohol	38 (67.9)	29 (52.7)	36 (63.2)	0.25
Hypertension	34 (60.7)	19 (34.6)^†^	16 (28.1)	0.0010
Antihypertensive treatment	21 (37.5)	12 (21.8)	6 (10.5)	0.0030
Diabetes mellitus	5 (8.9)	4 (7.3)	0 (0.0)*	0.081
Mean (SD) of characteristic				
Age, years	51.9 (14.1)	47.9 (13.0)	39.0 (14.7)^†^	<0.0001
BMI, kg/m^2^	28.2 (5.0)	27.1 (4.8)	25.7 (4.0)	0.015
Systolic pressure, mmHg	130.9 (17.0)	123.4 (12.3)^†^	125.4 (14.0)*	0.022
Diastolic pressure, mmHg	83.8 (11.6)	80.4 (10.3)	78.2 (11.0)	0.025
Heart rate (beats per minute)	60.5 (10.4)	63.4 (9.4)	62.0 (10.6)	0.33
Serum total cholesterol, mmol/L	5.06 (0.81)	5.24 (0.83)	5.13 (0.88)	0.54
Serum HDL cholesterol, mmol/L	1.46 (0.34)	1.44 (0.34)	1.48 (0.36)	0.85
Plasma glucose, mmol/L	5.09 (0.97)	4.93 (0.77)	4.79 (0.40)	0.10
Serum creatinine, µmol/L	83.1 (17.1)	78.6 (16.4)	80.2 (13.9)	0.32
CRAE, µm	148.1 (16.7)	152.5 (13.5)	154.6 (14.7)	0.065
CRVE, µm	215.5 (20.3)	219.0 (17.9)	221 (18.3)	0.30
AVR	0.69 (0.07)	0.70 (0.06)	0.70 (0.06)	0.51
Geometric mean (IQR) of characteristic				
γ-glutamyltransferase (units/L)	24.8 (15.0–36.0)	22.8 (17.0–33.0)	22.1 (11.0–30.0)	0.33

Abbreviations: SD, standard deviation; LTL, leukocyte telomere length; BMI, body mass index; HDL, high-density lipoprotein; CRAE, central retinal arteriolar equivalent; CRVE, central retinal venular equivalent; AVR, arteriole-to-venule ratio; IQR, interquartile range. Significance of the differences with the left adjacent group: *P ≤ 0.05, ^†^*P* ≤ 0.01.

### Determinants of LTL and the retinal microcirculation

A 1-year increment in age was associated with a −0.40% (95%CI: −0.64 to −0.16%, *P* = 0.0012) and a −0.66% (95%CI: −0.92 to −0.41%, *P* < 0.0001) shorter LTL in women and men respectively. Pearson correlation plots between age (with indications of age quartiles) and baseline LTL measures of the total population (r = −0.40, *P* < 0.0001) and for men (r = −0.47, *P* < 0.0001) and women (r = −0.32, *P* = 0.0017) separately are provided in Supplementary Fig. S1. After adjustment for age a 1-kg/m^2^ increase in BMI was associated with −0.76% (95%CI: −1.31 to −0.20%, *P* = 0.0081) shorter LTL. We observed that age adjusted LTL in men, decreased with −0.64% (95%CI: −1.51 to 0.25%, *P* = 0.16) and in women decreased with −0.88% (95%CI: −1.55 to −0.22%, *P* = 0.010) for a 1-kg/m^2^ increase in BMI. Age-adjusted LTL was −2.22% (95%CI: −7.38% to 3.22%, *P* = 0.41) in women compared with men.

Among the 85 participants with 2 LTL evaluations, 58 (68.2%) showed telomere shortening and telomere attrition was associated with the duration between baseline and follow-up evaluation of LTL (Supplementary Fig. S2). LTL at baseline and follow-up were strongly correlated (r = 0.79, *P* < 0.0001) (Supplementary Fig. S3).

In women a 1-year increment in age resulted in a decrease of −0.26 µm (95%CI: −0.44 to −0.09 µm, *P* = 0.0036) in CRAE and was not associated with CRVE (*P* = 0.20). In men, the observed decrease in CRAE and CRVE for a 1-year increment in age was −0.46 µm (95%CI: −0.65 to −0.37 µm, *P* < 0.0001) and −0.42 µm (95%CI: −0.67 to −0.17 µm, *P* = 0.0013) respectively. AVR was not associated with chronological age in women and men separately but each year increase in age for the total population showed a negative trend of −0.0005 (95%CI: −0.001 to 0.000, *P* = 0.067) in the AVR. Age-adjusted CRAE was 6.0 µm (95%CI: 2.1 to 9.9 µm, *P* = 0.0028) wider in women compared with men. The age-adjusted AVR was −0.016 (95%CI: −0.031 to 0.000, *P* = 0.06) lower in men compared with women. No association was observed between age-adjusted CRVE and sex.

### Association between retinal microvasculature phenotypes and LTL

CRAE and AVR models including the three-way interaction term of sex × age × LTL showed to be significant (*P*-interaction = 0.037 and *P*-interaction = 0.028 respectively). CRAE and AVR models including the three-way interaction term of sex × BMI × LTL also showed to be significant (*P*-interaction = 0.010 and *P*-interaction = 0.010 respectively). Estimates for the association between CRAE, CRVE, AVR and LTL (Table 2) are therefore provided for men and women separately and for two different age or BMI values (based on the 25th and 75th percentile of age or BMI) respectively. The interaction terms sex × age × LTL or sex × BMI × LTL in CRVE models were not significant and no association between CRVE and LTL was observed in men nor women or for the entire population (data not shown).

**Table 2:** Association between retinal diameters and leukocyte telomere length, stratified by sex, age or BMI.

Outcome	Age^c^	Model	Men (*n* = 124)	Women (*n* = 129)	*P-*Interaction sex × age × LTL
			Estimate (95% CI)	Estimate (95% CI)	
CRAE^a^	33.5	Model1	−0.71 (−3.78, 2.35)	−1.39 (−4.64, 1.86)	0.037
	57.6	Model1	−4.57 (−7.25, −1.89)^†^	−0.07 (−2.95, 2.80)	
	33.5	Model2	−0.44 (−3.16, 2.29)	−1.14 (−3.97, 1.69)	0.018
	57.6	Model2	−4.30 (−6.67, −1.93)^†^	0.18 (−2.36, 2.72)	
CRVE^a^	33.5	Model1	−1.10 (−5.24, 3.05)	0.51 (−4.01, 5.03)	0.62
	57.6	Model1	−0.88 (−4.53, 2.78)	–0.92 (−4.84, 2.99)	
	33.5	Model3	−0.60 (−4.42, 3.22)	1.22 (−2.88, 5.32)	0.23
	57.6	Model3	1.31 (−2.11, 4.73)	−0.61 (−4.19, 2.98)	
AVR^b^	33.5	Model1	−0.001 (−0.015, 0.013)	−0.004 (−0.019, 0.010)	0.028
	57.6	Model1	−0.020 (−0.032, –0.008)^†^	0.002 (−0.012, 0.015)	
**Outcome**	**BMI** ^c^	**Model**	**Estimate (95% CI)**	**Estimate (95% CI)**	***P*** **-Interaction sex × BMI × LTL**
CRAE^a^	23.8	Model1	−1.54 (−4.21, 1.14)	−0.73 (−3.62, 2.15)	0.010
	29.9	Model1	−5.88 (−8.64, –3.11)	0.42 (−2.26, 3.09)	
	23.8	Model2	−1.36 (−3.73, 1.02)	−1.14 (−3.67, 1.38)	0.0030
	29.9	Model2	−5.07 (−7.52, −2.61)^‡^	0.78 (−1.56, 3.13)	
CRVE^a^	23.8	Model1	−0.49 (−4.10, 3.12)	1.15 (−2.84, 5.14)	0.74
	29.9	Model1	−2.94 (−6.75, 0.87)	−0.34 (−4.04, 3.36)	
	23.8	Model3	0.38 (−2.96, 3.73)	1.94 (−1.69, 5.58)	0.49
	29.9	Model3	−0.19 (−3.78, 3.40)	−0.47 (−3.83, 2.90)	
AVR^b^	23.8	Model1	−0.006 (−0.018, 0.006)	−0.008 (−0.021, 0.005)	0.010
	29.9	Model1	−0.019 (−0.032, −0.007)^†^	0.004 (−0.008, 0.016)	

Abbreviations: BMI, body mass index; CI, confidence interval; LTL, leukocyte telomere length; CRAE, central retinal arteriolar equivalent; CRVE, central retinal venular equivalent; AVR, arteriole-to-venule ratio. Model 1 adjusted for age, sex, systolic blood pressure, smoking status, BMI as fixed effects and adjusted for participants nested within a family cluster as random effect. Model 2 adjusted according to Model 1 with additional adjustment for CRVE; Model 3 adjusted according to Model 1 with additional adjustment for CRAE; ^a^Estimates presented as difference in arteriolar or venular diameter (μm) for a 20% shorter LTL. ^b^Estimates presented as difference in arteriole-to-venule ratio for a 20% shorter LTL; ^c^age in years and BMI in kg/m^2^ provided for the 25th and 75th percentile. Significance for estimates: ^†^*P* ≤ 0.01, ^‡^*P* < 0.0001.

**Figure 1:**
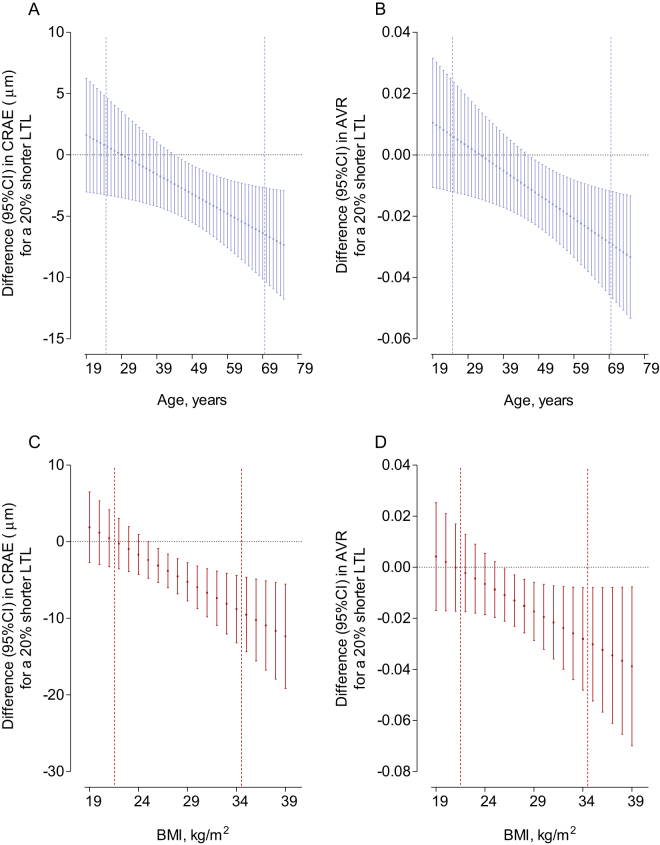
Effect size of the association of CRAE and AVR with average relative leukocyte telomere length (LTL) in men according to the age range (**A** and **B**) and BMI range (**C** and **D**). Effects presented as a difference in arteriolar (CRAE) diameter in μm or as the difference in arteriole-to-venule ratio (AVR) for a 20% shorter LTL. Models adjusted for age, sex, systolic blood pressure, smoking status, BMI as fixed effects and adjusted for participants nested within a family cluster as random effect. Dashed vertical lines indicates boundaries of 5th–95th percentile of age or BMI.

In men aged 57.6 years (75th percentile), a 20% shorter LTL was associated with a decrease of −4.57 µm (95%CI: −7.25 to −1.89 µm, *P* = 0.0011) in CRAE and a decrease of −0.020 (95%CI: −0.032 to −0.008 µm, *P* = 0.0018) in AVR. The association was observed from an age of 44 and 46 years onwards for the CRAE and AVR models respectively (Fig. 1A and B). Besides an effect modification of age on the association between CRAE and AVR and LTL in men, we observed also a BMI effect modification.

For men with a BMI of 29.9 kg/m^2^ (75th percentile), a 20% shorter LTL was associated with a smaller arteriolar diameter of −5.88 µm (95%CI: −8.64 to −3.11 µm, *P* < 0.0001), whereas at a BMI lower than 25 kg/m^2^ the association of LTL with CRAE was not observed (Fig. 1C). A 20% shorter LTL was associated with a decrease of −0.019 (95%CI: −0.032 to −0.007 µm, *P* = 0.0032) in AVR for men with a BMI of 29.9 kg/m^2^. No association between LTL and AVR was observed in men who had a BMI lower than 26 kg/m^2^ (Fig. 1D). Adjusting the CRAE models additionally for CRVE, did not influence the association between CRAE and LTL in men modified by age or BMI. Sensitivity analyses (Table 3) in which we excluded former and current smokers, or participants that had hypertension or had diabetes were consistent with the main models.

**Table 3:** Sensitivity analyses for the association of CRAE and AVR with leukocyte telomere length, stratified by sex and BMI.

Outcome	BMI^c^	Men		Women		*P-*Interaction sex × BMI × LTL
		*n*	Estimate (95% CI)	*n*	Estimate (95% CI)	
**CRAE** ^**a**^						
Excluding current and former smokers	23.6	58	−1.40 (−4.65, 1.86)	62	0.04 (−4.12, 4.20)	0.033
	29.4	58	−7.10 (−11.16, −3.03)^†^	62	1.02 (−3.56, 5.61)	
Excluding hypertension	23.1	56	−0.90 (−4.96, 3.15)	87	−1.23 (−4.99, 2.54)	0.0098
	28.5	56	−8.80 (−13.50, −4.10)^†^	87	−0.87 (−4.20, 2.45)	
Excluding diabetes	23.8	119	−1.50 (−4.17, 1.17)	123	−0.87 (−3.79, 2.05)	0.006
	29.3	119	−5.80 (−8.59, −3.02)^‡^	123	0.36 (−2.26, 2.97)	
**AVR** ^**b**^						
Excluding current and former smokers	23.6	58	−0.004 (−0.018, 0.010)	62	−0.006 (−0.024, 0.011)	0.082
	29.4	58	−0.015 (−0.032, 0.002)	62	0.006 (−0.014, 0.026)	
Excluding hypertension	23.1	56	−0.002 (−0.021, 0.018)	87	−0.006 (−0.023, 0.012)	0.036
	28.5	56	−0.029 (−0.052, −0.007)*	87	−0.002 (−0.018, 0.014)	
Excluding diabetes	23.8	119	−0.006 (−0.018, 0.007)	123	−0.008 (−0.022, 0.005)	0.0074
	29.3	119	−0.020 (−0.033, −0.007)^†^	123	0.003 (−0.009, 0.015)	

Abbreviations: BMI, body mass index; CI, confidence interval; LTL, leukocyte telomere length; CRAE, central retinal arteriolar equivalent; AVR, arteriole-to-venule ratio. Models adjusted for age, sex, systolic blood pressure, smoking status, BMI as fixed effects and adjusted for participants nested within a family cluster as random effect. ^a^Estimates presented as difference in arteriolar diameter (μm) for a 20% shorter LTL; ^b^Estimates presented as difference in arteriole–to–venule ratio for a 20% shorter LTL; ^c^BMI in kg/m^2^ provided for the 25th and 75th percentile. Significance for estimates: *P ≤ 0.05, ^†^*P* ≤ 0.01, ^‡^*P* < 0.0001.

## Discussion

The main finding of this study is that we observed a sex-specific association between retinal microvascular traits reflected by CRAE, AVR and LTL, which was dependent on age and BMI. More specifically, in older aged or overweight men, smaller retinal arteriolar diameters and a decrease in the arteriolar to venule ratio were associated with biological age as represented by a shorter LTL when compared with younger aged or normal weight men. No associations were observed between retinal microvascular traits and LTL in women.

After searching for studies in MEDLINE using the search terms “telomere length”, “retinal diameters”, “retinal microvascular”, this is to our knowledge the first report on an a significant association between retinal microvascular phenotypes and telomere length in a general population. In a cross-sectional study of 216 South African HIV-infected adults, an inverse relation between venular diameters and telomere length was observed^16^. The same authors report, in an age, sex and socio-economic matched HIV-uninfected counter part of the HIV-infected population, no association between the diameter of retinal arterioles and telomere length, although a positive trend was observed (*P* = 0.15)^17^. These studies did not report a stratification for sex and may not be representative for the general population at large due to participants selection bias.

We did only observe an association between CRAE and AVR and LTL in men but not in women. These results are in line with the observation that telomere length in women are longer compared with men^18^, and that these longer telomere lengths in women are proposed as a potential underlying mechanism explaining why women may have a longer lifespan and lower risk for cardiovascular disease^19^. In the FLEMENGHO study LTL in women showed to be longer compared with LTL men^20^. Although in this subset of the population LTL did not differ in men compared with women, but we found however a stronger inverse association between chronological age and LTL in men compared with women. The sex difference in telomere length is suggested to be attributed firstly by the active properties of estrogens which are more highly produced in women from puberty onwards and secondly by the heterogametic disadvantage theory and a possible X-linked inheriting component^21–23^.

Estrogens may provide a high protective capacity in women as it possesses antioxidative functions as well as it may enhance telomerase (telomere elongation enzyme) activity^24^. The antioxidative functions such as scavenging free radicals and regulating antioxidant enzyme expression^25^ may lead to reductions in the formation of reactive oxygen species (ROS) which otherwise can lead to the production of single strand DNA breaks. Telomeres are highly rich guanine containing stretches, which have a high oxidation potential and therefore are highly vulnerable to ROS, leading to increased formations of 8-oxoguanines (8-oxoG). These 8-oxoG lesions lead to DNA breakage in telomeres which are less repaired compared with other parts of the genome^26^. In our study, we did not observe a difference in age-adjusted LTL between post-menopausal and pre-menopausal women. Besides also no difference in age-adjusted LTL was observed between women indicating the use of estrogen containing contraceptives or estrogen containing hormone therapy and women indicating not to use any estrogen containing agents (data not shown). A potential reason for not observing an association between LTL and menopausal status may be due to the small amount of women in the pre-menopausal (n = 52, 58.4%) and post-menopausal (n = 37, 41.6%) period during the baseline evaluation in this study. Besides we were not able to evaluate actual measured estrogen levels in relation with LTL, which may provide a more accurate actual hormonal status of the women. In this regard, recently Dålgard *et al*., unexpectedly even observed a higher LTL attrition rate in pre-menopausal women compared with post-menopausal women, indicating that not alone estrogen may explain the sex-related LTL characteristics^27^.

Therefore aside from the role of estrogen, other sex-specific factors may explain our observed effects. A higher general oxidative stress status in men compared with women may account to some extent for the sex-specific associations observed. As reviewed recently, women exhibit a lower oxidative stress level compared with men, due to a lower ROS production, lower presence of oxidative stress biomarkers and a higher antioxidant potential^28^. For instance Ide and colleagues showed that in young men oxidative stress related plasma thiobarbituric acid-reactive substances and urinary 8-isoprostaglandin F_2α_ concentrations were significantly higher compared with age-matched women, independent from endogenous antioxidant enzymes, vitamin E, or estrogens^29^. In addition to oxidative stress differences, the genetic background in men compared with women may potentially explain sex-specific ageing differences. Asymmetric inheritance has led to the heterogametic sex-hypothesis indicating that due to the presence of two X chromosomes in women, the phenotypic effect of sex-linked deleterious alleles are masked by normal alleles on the other X. In men, no second X chromosome is available to compensate for a single adverse allele^30^. In addition to this, also the asymmetric inheritance of the mitochondrial genome has indicated a more optimal mitochondrial function in women compared with men^31^.

We observed stronger associations of improved retinal microvasculature with biological age with increasing age and BMI in men. Whether age or BMI is the major or independent modifier of the association between retinal outcomes and LTL in men could not be fully elucidated. This because of the high correlation between the two different three-way interaction terms. CRAE models including both the three-way interaction terms (sex × age × LTL and sex × BMI × LTL) showed no interaction neither for age (*P* = 0.22) nor BMI (*P* = 0.10). Although age is not a modifiable factor, and our results shows independent of age stronger associations between telomere length and CRAE at higher BMI, this suggests that maintaining a healthy weight for men is beneficial for a healthy microvascular condition.

A 1-year increase of chronological age in men showed a decrease in CRAE of 0.46 µm. Comparable chronological age effects on CRAE have been observed in other study populations. In 4,247 participants (mean age of 61 years) of the Beaver Dam Eye Study, each year of chronological age was associated with a decrease in CRAE of 0.21 µm (95%CI: −0.15 to −0.27 µm, *P* < 0.001) with stronger effects observed in men^32^. In another Flemish population of 84 participants (mean ± SD age of 37 ± 9 years) a 1-year age effect showed a decrease in CRAE of 0.59 μm (95% CI: −0.94, −0.23, *P* = 0.0015)^33^. For a public health comparison we compared the LTL estimates on CRAE with the estimates of chronological age observed in our study. Our observed effect-size of a 20% decrease in LTL on CRAE corresponds to an estimated chronological age effect of approximate 8.7 to 13.0 years.

In the FLEMENGHO cohort we were able to examine telomere attrition in 85 participants. We observed a strong association between LTL measured at baseline and follow-up, which is in line with the observation that persons have a clear telomere tracking and ranking across the lifespan. This indicating that persons having long telomeres at a certain time point will persist having long telomeres at a different time point in their life^34,35^. Additionally we observed that the attrition rate depended on the LTL at baseline, indicating that a long LTL at baseline implies a higher attrition rate, which has been observed in other population studies and potentially relies on epigenetic regulations of telomere maintenance or the preferentially action of telomerase on short telomeres^36–38^. Lastly, we need to note that we observed lengthening of telomeres in 31.8% of the participants. This observation is in line with increasing reports of prospective follow-up studies observing indeed lengthening of telomeres in participants ranging from 1.5% up to 50%^39,40^. Different possibilities have been proposed to explain this phenomenon. Firstly the main reason for this observation is attributed by measurement error, and it has been shown that lengthening of LTL has less frequent been observed with the Southern blot method (less laboratory error) compared to the qPCR method (more laboratory error)^41^. In this regard our qPCR showed an acceptable CV of 8.8%. Secondly, the follow-up period in studies may not be long enough to capture telomere shortening. Indeed it has been shown that a longer time between baseline and follow-up results in a lower percentage of participants showing LTL lengthening^39^. Thirdly, some biological mechanisms have been suggested to be involved in potential lengthening of LTL, including telomere dynamics in hematopoietic stem cells by which for instance inflammation may induce telomerase activity in lymphocytes or induce a clonal expansion of subpopulations of lymphocytes with longer telomeres^41–43^. Although the contribution of these factors are estimated to be relatively small.

This study needs to be interpreted within the context of its limitations. First, our sample size was relatively small, and as the follow-up rate was only 50.3%, we could not evaluate our data in a longitudinal fashion (n = 85), by which baseline LTL may be studied as a predictor for arteriolar narrowing. Nevertheless we found a strong association between retinal traits and LTL in men, even after excluding participants with increased risks for cardiovascular diseases in several sensitivity analyses, although a causal relation cannot be assessed in this study. Second, our population consists only of white-Europeans, and both retinal microvasculature and telomere length may be associated with ethnic or racial differences. For instance the age and sex adjusted mean arteriolar diameters were smaller in a population of 215 African Carribeans compared with 323 White Europeans (17.90 vs 18.36 pixels)^44^. Besides, age-and sex-adjusted T/S ratio has been showed to be greater in African-American adolescents compared with Caucasian adolescents (T/S ratio, 1.32 vs 1.27, P = 0.014)^45^. Given this, our results are less generalizable to other ethnicities and our findings should be further evaluated in other ethnic groups. Third, within this population we were not able to address the potential role of LTL as a mediator of obesity related inflammation on retinal outcomes as a potential underlying mechanism of the observed association. The role of inflammatory markers in relation to the association between LTL and microvascular phenotypes should be further evaluated. Additionally we acknowledge that the potential confounding of other unmeasured or unidentified factors associated with ageing cannot be ruled out. Nevertheless we were able to exclude some potential confounding factors in sensitivity analyses by excluding hypertensive participants, smokers or diabetic patients.

In conclusion, a poorer retinal microvasculature is associated with biological age in men. The association of retinal microvascular traits and LTL were more pronounced with increasing age and BMI in men. In women, retinal microcirculation was not associated with LTL, suggesting a potential protective mechanism against oxidative stress and inflammation which potentially may be explained by the function of estrogens. As having a severe microvasculature phenotype may underlie increased risks in target organ damage and an earlier onset in the development of cardiovascular incidences and the development of diabetes mellitus, our results are in line with current recommendations that maintaining a healthy weight and lifestyle may be associated with a slower ageing both at the organ (microcirculation) and molecular (telomere length) level.

## Methods

### Study population

This study is nested within the family-based population study FLEMENGHO (Flemish Study on Environment, Genes and Health Outcomes). Recruitment started in 1985 and continued until 2004. Study participants are representative for a geographically defined area in Northern Belgium. The Ethics Committee of the University of Leuven approved the FLEMENGHO protocol^46^. The FLEMENGHO study has been carried out according to the Helsinki declaration^47^. Participants gave informed written consent. The initial participation rate was 78.0%. The participants were repeatedly followed up^46^. At each contact, standardized questionnaires were completed to collect detailed information about each participant’s personal and familial medical history, use of medication, smoking habits, intake of alcohol, and lifestyle. From January 2008 onwards, high-fidelity phenotyping at the local examination center in the catchment area included retinal photography. The participation rate for initial retinal photography amounted to 76.0% and resulted in 746 participants with retinal photography at baseline (between January 2008 and March 2013). Of these 746, we excluded 578 participants because no high quality DNA was available for LTL assessment at the same occasion, therefore this study included 168 participants with retinal photographs and a LTL measurement available at baseline. As our subset included participants evaluated in a time period of 5.25 years, we achieved to have a follow-up examination of these traits in only 85 participants, which had a median interval between baseline and follow-up of 4.1 (range: 2.7 to 4.8) years. The number of participants available for statistical analysis therefore totalled 253.

### Retinal photography

Participants were asked to refrain from heavy exercise, smoking and drinking alcohol or caffeine-containing beverages for at least 3 h before retinal imaging. We applied a non-mydriatic approach in a dimly lit room to obtain retinal photographs, one image per eye in each participant, with the Canon Cr-DGi retinal visualization system combined with the Canon D-50 digital camera (Canon Inc, Medical Equipment Group, Utsunomiya, Japan). Two trained observers applied the validated computer-assisted program IVAN (Vasculomaticala Nicola, version 1.1, Department of Ophthalmology and Visual Science, University of Wisconsin-Madison, Madison, WI, USA) based on formulae published by Parr^48^ and Hubbard^49^. The software returns the average retinal arteriolar and venular diameters according to the revised Knudtson formula^50^. The retinal microvascular diameters are expressed as central retinal arteriolar equivalent (CRAE), central retinal venular equivalent (CRVE) and their ratio (AVR). For analysis, measurements of right and left both eyes were averaged. Intra-observer variability according to the Bland and Altman method^51^ was 11.7% for CRAE, 9.6% for CRVE, and 12.5% for AVR^52^. The corresponding estimates for inter-observer variability were 10.8%, 9.9%, and 14.6%^52^.

### Average relative LTL measurement

Peripheral blood was collected from each participant. DNA was extracted from buffy coat using the QIAamp DNA Mini Kit (Qiagen, Inc., Venlo, the Netherlands). Relative average LTL was assessed as described previously^53^ by the use of a modified quantitative real-time PCR (qPCR) protocol. In brief, for each sample in triplicate, the telomeric region was amplified with the use of telomere specific primers (telg and telc) and one single-copy gene was amplified (*36B4*) on a 7900HT Fast Real-Time PCR System (Applied Biosystems, City, Country) in a 384-well format. Cycle thresholds after the amplification of the telomere specific region were normalized relative to the cycle thresholds after the amplification of the single-copy gene using the qBase software (Biogazelle, Zwijnaarde, Belgium). Relative average leukocyte telomere were expressed as the ratio of telomere copy number to single copy gene number (T/S) relative to the average T/S ratio of the entire sample set. Reaction efficiency was assessed on each reaction plate (using a 6-point serial dilution of pooled buffy coat DNA) and inter-run calibrators were used to account for inter-run variability. We achieved coefficients of variation (CVs) of 0.67%, 0.41% and 8.8% for telomere runs, single-copy gene runs and T/S ratios, respectively.

### Other measurements

On each examination day, trained nurses measured the participants’ blood pressure and anthropometric characteristics. Blood pressure was the average of five consecutive auscultatory readings obtained with a standard mercury sphygmomanometer according to European guidelines. Hypertension was a blood pressure of at least 140 mm Hg systolic or 90 mm Hg diastolic or use of antihypertensive drugs. Body mass index (BMI) was weight in kilograms dived by the square of height in meters. After participants had been fasting for at least 6 hours, venous blood samples were drawn. Plasma glucose, serum levels of total and high-density lipoprotein (HDL) cholesterol, creatinine and γ-glutamyltransferase (as a biomarker of alcohol intake) were measured using automated methods in a single certified laboratory. Diabetes mellitus was a self-reported diagnosis, a fasting glucose level exceeding 7.0 mmol/L (126 mg/dL) or the use of antidiabetic agents^54^.

### Statistical analysis

Data base management and statistical analyses were performed, using SAS 9.3 software (SAS Institute Inc., Cary, NC, USA). Normality of the data was tested using the Shapiro-Wilk test of normality. LTL was normalized by a logarithmic (log_10_) transformation. To study the possible confounding structure of our dataset, we used ANOVA and χ^2^ statistics for comparing the distribution of means and proportions respectively across tertiles of LTL. We used general linear mixed models to associate the retinal measurements with LTL, while accounting for family cluster and individuals as random effect. Models were adjusted for sex, age, systolic blood pressure, body mass index and smoking status as a fixed effect. We tested the three-way interactions sex × age × LTL and sex × BMI × LTL in separate models. In fully adjusted analyses, CRAE models were additionally adjusted for CRVE and vice versa. Model estimates are presented as a difference in arteriolar or venular diameter in µm (CRAE and CRVE models) or as a difference in arteriole-to-venule ratio (AVR models) for each 20%-decrease in LTL. As sensitivity analyses, we excluded current and former smokers or patients with hypertension or diabetes mellitus in the models containing the sex × BMI × LTL interaction.

### Data availability statement

The datasets generated during the current study are available from the corresponding author on reasonable request.

## Electronic supplementary material

Supplementary Material
