# Supplementary material for: Retinal microcirculation and leukocyte telomere length in the general population
Source: Sci Rep. 2018 May 4;8:7095. doi: 10.1038/s41598-018-25165-6 (PMC5935741; doi:10.1038/s41598-018-25165-6)
Supplement: Supplementary file 1 — Supplementary Material [file 41598_2018_25165_MOESM1_ESM.docx]

**Supplementary Material to:**

**Retinal microcirculation and leukocyte telomere length
in the general population**

Dries S Martens,^1^ Fang-Fei Wei,^2^ Bianca Cox,^1^ Michelle Plusquin,^1^ Lutgarde Thijs,^2^
Ellen Winckelmans,^1^ Zhen-Yu Zhang,^2^ Tim S Nawrot,^1,3^ Jan A Staessen^2,4^

^1^Centre for Environmental Sciences, Hasselt University, Hasselt, Belgium
^2^Studies Coordinating Centre, Research Unit Hypertension and Cardiovascular Epidemiology, KU Leuven Department of Cardiovascular Sciences, University of Leuven, Leuven, Belgium
^3^Department of Public Health & Primary Care, University of Leuven, Leuven, Belgium
^4^R&D Group VitaK, Maastricht University, Maastricht, The Netherlands.

**Supplementary Figure S1. Pearson correlation between age (years) and LTL (T/S ratio) at baseline.** Correlations showed for the entire population (A), men (B) and women(C) separately. Dashed vertical lines represents from left to right the 25th, 50th and 75th percentile of the age distribution.

**Supplementary Figure S2. Telomere attrition characteristics for 85 participants with a follow-up.** (A) Pearson correlation between telomere attrition (ΔLTL, as defined by LTL at baseline minus LTL at follow-up) and duration (years) between baseline and follow-up. (B) Telomere shortening or lengthening for each individual separately compared with LTL set at 0 at baseline as reference.

**Supplementary Figure S3. Pearson correlation between LTL at baseline and follow-up.**
